# Prevalence of hearing loss among patients with type 2 diabetes

**DOI:** 10.25122/jml-2021-0300

**Published:** 2022-06

**Authors:** Mohammadreza Shafiepour, Zahra Bamdad, Masoud Radman

**Affiliations:** 1 Department of Internal Medicine, Rafsanjan University of Medical Sciences, Rafsanjan, Iran; 2 Department of Otolaryngology, Faculty of Medicine, Rafsanjan University of Medical Sciences, Rafsanjan, Iran

**Keywords:** hearing loss, diabetes mellitus, type 2, pure tone audiometry

## Abstract

The auditory pathway is the main target for high levels of blood sugar. Increased glucose in diabetic patients can disrupt the auditory system physiologically and anatomically. The present study aimed to examine the prevalence of hearing loss in patients with type 2 diabetes. A total of 94 patients with type 2 diabetes, aged from 40 to 80 years, were selected randomly in the present descriptive cross-sectional study for pure tone audiometry (PTA), speech discrimination score (SDS), and speech reception threshold (SRT) tests. Accordingly, patients with a hearing threshold larger than or equal to 25 dB were considered hearing-impaired according to the PTA test. In addition, the patients' speech discrimination score was measured using a list of monosyllabic words with an intensity of 40 dB or more than the SDS test. However, in the SRT test, the patients' superficial speech comprehension threshold was measured using a list of two-syllable words. Most diabetic patients had hearing loss in both right and left ears based on the PTA and SRT tests. However, they did not have hearing loss in both ears according to the SDS test. There was no correlation between the PTA, SRT, and SDS tests and blood creatinine levels in both ears (p>0.05). Nevertheless, the right ear had a positive correlation with systolic blood pressure only in the PTA test (p<0.05). The difference between the two groups of men and women with type 2 diabetes in the hearing level in the right and left ears was not statistically significant. Hearing loss is a common deficiency in diabetic patients. In addition, it seems that diabetes is an independent risk factor for the hearing loss level.

## INTRODUCTION

Hearing loss is one of the most common disabilities in the United States. The prevalence of this defect is more than 16% in the adult population and two-thirds of the population over 70 [1]. Hearing loss is associated with controllable factors, such as ototoxic medicines, exposure to loud tones and noise, and uncontrollable factors, including age, sex, and genetics [2]. The role of chronic diseases in the etiology of hearing disorders has recently turned into an interesting research subject [2]. Research shows that diabetes is associated with hearing loss and that two-thirds of diabetic adults in the United States suffer from hearing loss, limiting their ability to communicate with others [3–5].

The auditory pathway has high metabolic activity, so it is the target organ for the pathogenesis of hyperglycemia and diabetes [6]. High blood sugar leads to a number of metabolic disorders impairing the auditory system physiologically and anatomically [7]. Diabetes complications, including coronary artery disease and nephropathy, have been linked to hearing loss. On the other hand, some studies reported the role of high levels of low-density lipoprotein (LDL), low levels of high-density lipoprotein (HDL), hypertension, smoking, central obesity, alcohol consumption, high triglycerides, blood sugar, and aging in hearing impairment in diabetic patients [3, 8, 9].

Several mechanisms have been suggested for the development of hearing loss in diabetic patients, including microangiopathy, neuropathy, and oxidative stress damage [10, 11]. Diabetes can cause some changes to the stria vascularis, the basement membrane, and the cochlear hair cells. The cochlea has a large number of small blood vessels exposed to microangiopathy during hyperglycemia [12]. Besides, hyperglycemia leads to oxidative stress (OS) and the production of free radicals, thereby leading to neuronal damage and microglial activation. These changes lead to the degeneration of the small blood vessels feeding the atrial-cochlear nerve responsible for hearing and balance [2, 13].

Bainbridge et al. (2016) examined risk factors for hearing loss in diabetic patients. Their results showed that alcohol consumption, smoking, chronic renal failure, and high triglycerides were significantly associated with hearing loss [3]. Nwosu et al. (2017) showed that the prevalence of hearing loss was higher in diabetic adults (46.9%) than in the control group (15.6%) [14]. Samelli et al. (2016) reported that the hearing threshold and auditory-verbal were 46.3 and 26.3% in diabetic patients and the control group, respectively [15].

The auditory pathway is susceptible to metabolic changes due to hyperglycemia, so diagnosing hearing impairment in diabetic patients is important. Thus, evaluating the prevalence of hearing loss in diabetic patients is effective in finding a way to decrease hearing loss among them. Therefore, this study aims to assess the prevalence of hearing loss in diabetic patients.

## MATERIAL AND METHODS

The target population in the present descriptive cross-sectional study was patients with type 2 diabetes referred to the endocrinology department and diabetes clinic of Ali Ibn Abi Taleb Hospital, Rafsanjan, in 2018. Accordingly, 9,206 patients entered this study. In the end, 94 patients with type 2 diabetes [16], aged from 40 to 80, were selected randomly for pure tone average (PTA), speech discrimination score (SDS), and speech reception threshold (SRT) tests [13]. The exclusion criteria consisted of patients with noisy jobs, a history of ear trauma and ototoxic drug use, congenital hearing loss [2], history of hearing loss before diabetes diagnosis, older than 80 years and younger than 40 years, and having a tympanic membrane examination.

The criteria for diagnosing diabetes in people were having a fasting blood sugar of more than 125 mg/dL and the glucose tolerance test (GTT) ≥200 or 6.5 ≤HbA1c [16]. The patient's blood pressure was checked using a pressure gauge cuff. To determine the blood creatinine level, the information in the patients' files, which was recorded at the last visit, was utilized. In addition, a physical examination of both ears was performed using an otoscope. Next, the patients with no tympanic membrane rupture were referred to the Moradi Hospital in Rafsanjan for audiometric tests.

Patients underwent audiometric testing using the PTA, SDS, and SRT tests. In the PTA test, patients were subjected to sounds at different frequencies, and their hearing thresholds of air transmission and bone transfer were measured separately using an audiometer. Accordingly, patients with a hearing threshold higher than or equal to 25 dB were considered to have hearing impairment in the PTA test. In addition, the patients' speech discrimination score was measured using a list of monosyllabic words with an intensity of 40 dB or higher in the SDS test. Finally, in the SRT test, the patients' superficial speech comprehension threshold was measured using a list of two-syllable words [17].

The data were analyzed using SPSS V21.0. The groups were compared using parametric and non-parametric statistical methods. Student's t-test, chi-square test, one-way ANOVA, and Tukey's post hoc test were used to compare the data between different groups. The data were presented as mean (SD). In addition, differences were considered statistically significant at P<0.05 and P<0.01.

## RESULTS

The patients' mean age was 61.54±10.16. The data showed that most patients (63 patients) were female and housewives; 25 were employees, 5 were workers, 14 were farmers, and 50 were housewives (Table 1). The creatinine level was normal in most patients with type 2 diabetes (95.7%) and those with mild hearing loss (35.1%). Moreover, the data showed that the systolic blood pressure was normal in 62.8% of the patients, and diastolic blood pressure was high in 93.6% of the patients. In addition, 63 and 31 patients received oral medicines and insulin, respectively. Therefore, most diabetic patients were administered oral medicines (Table 1).

**Table 1:** Distribution of patients with type 2 diabetes (n=94).

Parameters			Percentage
**Age**	≤65		69.1
	>65		30.9
**Gender**	Male		33
	Female		67
**Occupation**	Employee		26.6
	Worker		5.3
	Farmer		14.6
	Housewife		53.2
**Creatinine level**	<1.2		95.7
	>1.2		4.3
**Hearing loss level**	With hearing loss	Mild	35.1
		Moderate	26.6
		Severe	9.6
		Without hearing loss	28
**Systolic blood pressure**	Normal		62.8
	High		37.2
**Diastolic blood pressure**	Normal		6.4
	High		93.6
**Type of medicine**	Oral medicine		67
	Insulin		31

However, the results showed a significant difference in terms of the age of all groups and different hearing loss levels (p<0.01). Also, there was no significant difference between the two genders in terms of the prevalence of hearing loss (p>0.05) (Table 2). Creatinine levels in most diabetic patients were less than 1.2, and only 4 patients had high creatinine levels. Furthermore, there was no significant difference between patients with creatinine levels less and more than 1.2 in terms of the prevalence of hearing loss (p>0.05) (Table 2). Systolic blood pressure in most diabetic patients with mild hearing loss was higher than in other patients. However, patients with moderate, severe, and no hearing loss had normal systolic blood pressure. In fact, the difference between high and normal systolic blood pressure was statistically significant (p<0.01) (Table 2).

**Table 2:** Prevalence of hearing loss in patients with type 2 diabetes based on their age, gender, and creatinine level (n=94).

Parameters		With hearing loss			Without hearing loss	X^2^	P-value
		Mild	Moderate	Severe			
**Age**	<65	36.9%	18.5%	6.2%	38.5%	14.97	p<0.01
	≤65	31%	44.8%	17.2%	6.9%		
	Total	35.1%	26.6%	9.6%	28.7%		
**Gender**	Male	35.5%	25.8%	9.7%	29%	0.01	1.000
	Female	34.9%	27%	9.5%	28.6%		
	Total	35.1%	26.6%	9.6%	28.7%		
**Creatinine level**	<1.2	34.4%	27.8%	8.9%	28.9%	2.43	0.487
	1.2<	50%	0%	25%	25%		
	Total	35.1%	26.6%	9.6%	28.7%		

There was no significant difference between high and normal diastolic blood pressure, as most of the diabetic patients showed mild, moderate, severe, and no hearing loss (36.4, 27.3, 8, and 28.4%, respectively), with high diastolic blood pressure (p>0.05). Besides, there was no significant difference between different types of medicines in terms of the prevalence of hearing loss (p>0.05) (Table 3). Most of the patients with mild and moderate hearing loss had a sensorineural hearing loss; the majority of the patients with severe hearing loss were deaf. Also, there was a significant difference between different types of hearing loss (p<0.01, X^2^=1.40) (Table 3).

**Table 3:** Prevalence of hearing loss in patients with type 2 diabetes based on their systolic and diastolic blood pressure, type of medicines, and type of hearing loss (n=94). P<0/01**.

Parameters		With hearing loss			Without hearing loss	X^2^	P-value
		Mild	Moderate	Severe			
**Systolic blood pressure**	Normal	25.4%	22%	11.9%	40.7%	14.22	0.003**
	High	51.4%	34.3%	5.7%	8.6%		
	Total	35.1%	26.6%	9.6%	28.7%		
**Diastolic blood pressure**	Normal	16.7%	16.7%	33.3%	33.3%	4.68	0.196
	High	36.4%	27.3%	8%	28.4%		
	Total	35.1%	26.6%	9.6%	28.7%		
**Drug types**	Oral	28.6%	30.2%	11.1%	30.2%	3.84	0.279
	Insulin	48.4%	19.4%	6.5%	25.8%		
	Total	35.1%	26.6%	9.6%	28.7%		
**Type of hearing loss**	Sensorineural	54.5%	40.9%	4.5%	0%	1.40	0.001**
	Conductive	100%	0%	0%	0%		
	Mixed	16.7%	58.3%	25%	0%		
	Deafness	0%	0%	80%	20%		
	Total	35.1%	26.6%	9.6%	28.7%		

According to the PTA test, most patients had hearing loss in both right and left ears (56.4 and 68.1%, respectively). Similarly, most of the patients had hearing loss in both right and left ears (67 and 84%, respectively), according to the SRT test. Nevertheless, the SDS test showed that most patients had no hearing loss in the right and left ears (97.9 and 91.5%, respectively) (Table 4). The right ear had a significant positive relationship with systolic blood pressure (p<0.05) only in the PTA test. However, there was no positive relationship between creatinine levels and diastolic blood pressure in the PTA, SRT, and SDS tests (Table 5).

**Table 4:** Prevalence of hearing loss in patients with type 2 diabetes based on PTA, SDS, and SRT tests (n=94).

Hearing loss level	PTA		SRT		SDS	
	Right ear	Left ear	Right ear	Left ear	Right ear	Left ear
**With hearing loss**	56.4%	68.1%	67%	84%	2.1%	8.5%
**Without hearing loss**	53.6%	31.9%	33%	16%	97.9%	91.5%

PTA – pure tone audiometry; SDS – speech discrimination score; SRT – speech reception threshold.

**Table 5:** Correlations between PTA, SRT, and SDS scores with blood creatinine levels as well as systolic and diastolic blood pressure (n=94).

Tests Parameters		PTA		SRT		SDS	
		Right ear	Left ear	Right ear	Left ear	Right ear	Left ear
**Creatinine level**	r	-0.15	0.08	-0.10	0.07	0.02	-0.17
	P-value	0.133	0.438	0.330	0.449	0.820	0.090
**Systolic blood pressure**	r	0.24*	0.13	0.19	0.19	-0.15	-0.07
	P-value	0.017	0.201	0.065	0.064	0.127	0.490
**Diastolic blood pressure**	r	-0.12	-0.003	-0.09	0.01	0.002	-0.05
	P-value	0.249	0.979	0.355	0.924	0.984	0.576

P<0.05*. PTA – pure tone audiometry; SDS – speech discrimination score; SRT – speech reception threshold.

**Figure 1:**
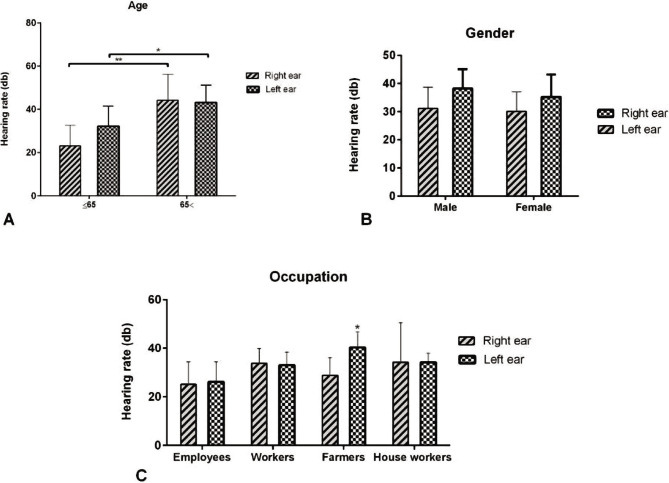
Comparison of hearing levels in patients with type 2 diabetes in terms of age, gender, and occupation. A – Hearing rate based on less or more than 65 years old in both right or left ears; B – Hearing rate based on gender (male or female) in both ears; C – Hearing rate based on people 's job in both right and left ears.

Younger patients aged less than 65 had significantly higher hearing levels in both right and left ears than patients older than 65 years (Figure 1 A). Female patients had higher hearing levels in both right and left ears than male patients, yet there was no significant difference in the right and left ear hearing levels (Figure 1 B). According to the PTA test, the average level of right ear hearing among housewives was higher than that of other patients, and employed patients had the lowest level among others. According to the results, the right ear hearing level was not significantly different, but the differences between the four groups of the patients with different occupations were statistically significant in the left ear hearing level (Figure 1 C).

## DISCUSSION

The results of the present study showed that most of the patients with type 2 diabetes (35.1%) had mild hearing loss. Besides, our findings showed that most patients with hearing loss were older than 65 years. In addition, patients under 65 years old had better hearing in both left and right ears. Furthermore, most of the patients with hearing loss were male. However, there was no significant difference between the two genders in the prevalence of hearing loss. Moreover, there was no significant difference between diabetic men and women in hearing loss in the right and left ears. Similarly, Ghanaati et al. reported no significant difference between males and females [18], with the findings of the two studies being consistent.

There was no significant difference in the prevalence of hearing loss between the two groups in the present study in terms of creatinine levels less than 1.2 and more than 1.2. Furthermore, the patients' scores in the PTA, SRT, and SDS tests in both right and left ears were not related to blood creatinine levels. Samelli et al. (2017) evaluated patients' scores in PTA, SDS, and SRT tests in the right and left ears and compared them with the mean of patients' blood creatinine levels. Their results showed no significant relationship between creatinine levels and hearing loss test scores in both ears, which is consistent with our study [15].

Bainbridge et al. (2016) evaluated risk factors in diabetic patients. Their findings showed that alcohol consumption, smoking, chronic renal failure, and high triglyceride levels were significantly associated with hearing loss in diabetic patients. In addition, low income was reported as a possible causative factor for hearing impairment [3]. Inconsistent with our findings, they reported chronic renal failure associated with increased creatinine levels and hearing loss. The difference between these two studies could be due to differences in the sample size. Accordingly, the study population was larger than our study, so the prevalence of people with renal insufficiency and high creatinine levels was higher.

The findings of the present study showed that systolic blood pressure was high in diabetic patients with mild hearing loss. Besides, diastolic blood pressure was high in diabetic patients with mild, moderate, and severe hearing loss as well as in those with no hearing loss. However, this difference was not statistically significant. Samelli et al. reported no association between the hearing threshold with age, gender, and hypertension. They concluded that the mean SRT and SDS in diabetic patients were 16.2±9.1% and 94.9±6.3%, respectively [15].

Mashhadi et al. (2015) examined the relationship between diabetes and hearing loss. Audiometric findings at different frequencies in diabetic and control groups showed no significant relationship between diabetes and hearing loss [19]. However, the relationship was more pronounced between hearing frequencies and patients' age. In addition, blood sugar control, diabetes duration, gender, microvascular complications (nephropathy, neuropathy, and retinopathy), and macrovascular complications (hypertension and cerebrovascular disease) were not related to hearing loss in diabetic patients [19]. Our findings were consistent with their study.

Consistent with our findings, Zivkovic-Marinkov et al. (2016) reported that the hearing threshold was significantly higher in diabetic patients than in healthy cases. Moreover, there was no difference among the patients in hearing loss in terms of different types of medications [20]. The results of the present study showed that diabetic patients had mild to moderate hearing loss. Nwosu et al. (2017) reported that the prevalence of hearing loss was 46.9 and 15.6% in diabetic patients and the control group, respectively [14]. The type of hearing loss was neurosensory which was consistent with our study.

Our study showed that mild to moderate hearing loss in diabetic patients was bilateral, with patients with a severe hearing loss having unilateral defects. Moreover, there was a significant difference between hearing loss types in diabetic patients. Accordingly, employed patients had better hearing in both right and left ears. In addition, housewives and employed patients had higher hearing loss in the right and left ear, respectively, than other patients, with no statistically significant difference. Hearing loss increased in farming patients, which could be due to their higher exposure to agricultural machines causing strong noise pollution.

## CONCLUSION

Our findings showed that 36.2% of diabetic patients suffered moderate to severe hearing loss. Also, there was a positive relationship between aging and hearing loss in diabetic patients. Accordingly, it seems that diabetes is a direct risk factor for hearing loss.
